# Annexin A4 Is Involved in Proliferation, Chemo-Resistance and Migration and Invasion in Ovarian Clear Cell Adenocarcinoma Cells

**DOI:** 10.1371/journal.pone.0080359

**Published:** 2013-11-11

**Authors:** Tae Mogami, Naho Yokota, Mikiko Asai-Sato, Roppei Yamada, Shiro Koizume, Yuji Sakuma, Mitsuyo Yoshihara, Yoshiyasu Nakamura, Yasuo Takano, Fumiki Hirahara, Yohei Miyagi, Etsuko Miyagi

**Affiliations:** 1 Molecular Pathology and Genetics Division, Kanagawa Cancer Center Research Institute, Yokohama, Japan; 2 Department of Obstetrics and Gynecology, Yokohama City University Graduate School of Medicine, Yokohama, Japan; The University of Hong Kong, China

## Abstract

Ovarian clear cell adenocarcinoma (CCC) is the second most common subtype of ovarian cancer after high-grade serous adenocarcinomas. CCC tends to develop resistance to the standard platinum-based chemotherapy, and has a poor prognosis when diagnosed in advanced stages. The *ANXA4* gene, along with its product, a Ca^++^-binding annexin A4 (ANXA4) protein, has been identified as the CCC signature gene. We reported two subtypes of ANXA4 with different isoelectric points (IEPs) that are upregulated in CCC cell lines. Although several *in vitro* investigations have shown ANXA4 to be involved in cancer cell proliferation, chemoresistance, and migration, these studies were generally based on its overexpression in cells other than CCC. To elucidate the function of the ANXA4 in CCC cells, we established CCC cell lines whose ANXA4 expressions are stably knocked down. Two parental cells were used: OVTOKO contains almost exclusively an acidic subtype of ANXA4, and OVISE contains predominantly a basic subtype but also a detectable acidic subtype. ANXA4 knockdown (KO) resulted in significant growth retardation and greater sensitivity to carboplatin in OVTOKO cells. ANXA4-KO caused significant loss of migration and invasion capability in OVISE cells, but this effect was not seen in OVTOKO cells. We failed to find the cause of the different IEPs of ANXA4, but confirmed that the two subtypes are found in clinical CCC samples in ratios that vary by patient. Further investigation to clarify the mechanism that produces the subtypes is needed to clarify the function of ANXA4 in CCC, and might allow stratification and improved treatment strategies for patients with CCC.

## Introduction

Ovarian cancer is the leading cause of mortality among gynecological malignancies in economically developed countries, with 100,300 new cases and 64,500 deaths in 2008 (GLOBOCAN 2008: http://globocan.iarc.fr/factsheet.asp). Epithelial ovarian carcinoma (EOC) is currently classified by its conventional clinical and histopathological features, together with recently uncovered molecular alterations, into five major subtypes: high-grade serous adenocarcinoma (HGSC), clear-cell adenocarcinoma (CCC), endometrioid adenocarcinoma, mucinous adenocarcinoma, and low-grade serous adenocarcinoma (LGSC) [1].

CCC is the second most common EOC subtype after HGSC. More than 50% of inactivating mutations in the AT-rich interactive domain 1A gene (*ARID1A*), almost 40% of activating mutations in the gene encoding the catalytic subunit of phosphatidylinositol 3-kinase (*PIK3CA*), and microsatellite instability characterize the genetic alterations in CCC [1]. A recent large-cohort study found a pattern in CCC of resistance to standard platinum-based chemotherapy, which offers poor prognosis for patients diagnosed in advanced clinical stages (i.e., International Federation of Gynecology and Obstetrics stages III and IV) [2]. The ratio of CCC among EOC in Japan is reported to be higher than that in Western countries (15–25% and 5–12%, respectively); ethnic or geographical differences in its incidence have also been noticed [3].

To understand the biological and clinical features of the four major EOC histotypes (LGSC and HGSC were defined as one subtype, SC) including CCC, using mRNA microarray analyses [4]–[9]. In these studies, CCC repeatedly showed characteristic gene signatures compared with other histotypes; many genes were picked up as the “CCC gene.” Schwartz et al. first showed expression of *ANXA4*, the gene encoding annexin A4 (ANXA4), to increase in CCC [4]. Zorn et al. found *ANXA4* again as an CCC gene, which is commonly up-regulated in clear cell adenocarcinomas derived not only in the ovary, but also in the endometrium and kidney [7]. Concurrently, proteomic-based studies were performed to illuminate CCC characteristics—especially, to find molecules involved in its inherent chemoresistance. We identified ANXA4 as an abundantly produced protein in human CCC cell lines compared with cell lines of other histotypes with two-dimensional fluorescent differential gel electrophoresis [10]. Although transcriptome analyses did not always find *ANXA4* as an CCC gene, recently, two strong studies that used proteomic and simultaneous immunohistochemical analyses of large numbers of clinical CCC specimens confirmed that ANXA4 is characteristically up-regulated in CCC [11], [12]. Two ANXA4 proteins with different isoelectric points (IEPs) were recognized using two-dimensional-polyacrylamide gel electrophoresis (2D-PAGE), and expression of both proteins was shown to be elevated in CCC cells [10], [12].

The annexins are ubiquitously expressed in most organisms, from fungi and protists, plants to animals. They contain multiple Ca^2+^-binding sites in the carboxyl-terminal region and exert diverse biological functions in a Ca^2+^-dependent manner, including vesicle trafficking, cell division, apoptosis, and growth control [13]–[15]. ANXA4 is one of 12 known vertebrate annexin proteins. It binds phospholipids in a Ca^2+^-dependent manner, self-associates as a trimeric complex on membrane surfaces, and is located in the nucleus, cytoplasm, or cell membrane [13], [15]. Increased expression of ANXA4 has been reported in various clinical epithelial tumors including gastric [16], colorectal [17], [18], pancreatic, breast, and laryngeal cancers [19] and a subset of ovarian SC [20], in addition to CCC and renal clear cell carcinoma [21]. Increased expression of *ANXA4* is associated with increased tumor stage and poorer patient prognosis in colorectal cancer [17], and with chemoresistance and poorer patient prognosis in ovarian SC [21].

*In vitro* functional analyses showed that forced overexpression of *ANXA4* induced carboplatin resistance in OVSAYO SC-cells [11], paclitaxel resistance in 293T cells [22], migration on vitronectin in MCF-7 breast cancer cells [21], and proliferation in AGS gastric cancer cells [16]. Few studies have investigated the function of ANXA4 directly in CCC cells. In the present study, to elucidate the function of highly expressed ANXA4 in CCC cells, we established CCC cell lines in which ANXA4 expression is consistently knocked down using *ANXA4* mRNA-targeting short hairpin RNA (shRNA) and investigated phenotypic changes such as cell proliferation, chemoresistance, and migration and invasion *in vitro*. We observed changes in these phenotypes in ANXA4 knockdown (KO) cells that suggest the two ANXA4 proteins with different IEPs have different function in CCC cells.

## Materials and Methods

### Cell lines and clinical samples

Two human ovarian clear cell adenocarcinoma cell lines, OVTOKO and OVISE, had been previously established and maintained in our laboratory [23], and also have been deposited in the JCRB Cell Bank (http://cellbank.nibio.go.jp/english/) for public use. Cells were cultivated at 37°C in a humidified atmosphere containing 5% CO_2_ unless otherwise described. Surgically removed tumor samples were frozen in liquid nitrogen and kept at −80°C or fixed in formalin and embedded in paraffin (FFPE samples), and used for 2D-PAGE or immunohistochemistry (IHC) respectively. Written informed consent was obtained from all the patients involved, and the institutional review board at Yokohama City University approved this study.

### Establishment of CCC cell lines with constitutively depressed ANXA4 expression

OVTOKO and OVISE cells were cultivated in RPMI-1640 medium supplemented with 10% fetal bovine serum (FBS; Invitrogen, Grand Island, NY, USA). Plasmid vectors to express shRNA that targeted *ANXA4* mRNA and a negative control non-specific shRNA were purchased from SuperArray Bioscience Corp. (KH06928N; Frederick, MD, USA). The oligonucleotide sequence to express each shRNA is 5′-GAGGGATGAAGGAAATTATCT-3′ for *ANXA4* and 5′-GGAATCTCATTCGATGCATAC-3′ for the negative control, respectively. Each shRNA-plasmid was introduced into OVTOKO and OVISE cells using Lipofectamine 2000 reagent (Invitrogen) according to the manufacturer's instructions. Stably transformed clones were selected under G-418 (Invitrogen) at 800 µg/mL and multiple independent cell lines were established and maintained for *in vitro* experiments.

### Evaluation of cell proliferation and chemo-sensitivity

Cell viability was evaluated by a WST-1 assay (Takara Bio Inc., Shiga, Japan) according to the manufacturer's instruction. All the cells maintained in G-418 were cultivated without G-418 for at least two passages before used in the following experiments. *ANXA4* knock down was confirmed just before use. Briefly, to test proliferation, cells were plated (1×10^4^ cells/well on 24-well plates) and cultivated for 4, 24, 48, 72, 96, or 120 h. Each sample was incubated for another 3 h with a WST-1 reagent, and the resultant formazan product in each well was read at 450 nm on a Wallac 1420 ARVO MX multi-label plate reader (PerkinElmer Inc., Waltham, MA, USA). To assess chemosensitivity, cells incubated for 24 h at 5×10^4^ cells/well on 24-well plates were subjected to varying concentrations of paclitaxel (0–100 µM) or carboplatin (0–75 µM). After 72 h, cell viability was evaluated as for the proliferation.

### Evaluation of cell migration and invasion activity

Cell migration and invasion activities were evaluated by a transwell assay with chambers separated with an 8 µm-pored membrane without (for migration assay) or with (invasion assay) Matrigel, using the BD BioCoa Tumor Invasion System (BD Bioscience, Franklin Lakes, NJ, USA) according to the manufacturer's instructions. Briefly, for the migration assay, 1×10^5^ cells were resuspended in 200 µl of RPMI-1640 with 0.1% bovine serum albumin (BSA), and added to the apical chamber. The basal chambers were filled with 500 µl of RPMI-1640 with different concentrations of FBS (0–5% for OVTOKO; 10% for OVISE). For the invasion assay, 2.5×10^5^ cells in 500 µl of RPMI-1640 with 0.1% BSA were added to the apical chamber, and the basal chambers were filled with 750 µl of RPMI-1640 with 10% FBS. After incubation (6 h for OVTOKO; 24 h for OVISE), non-invasive cells were removed from the apical chamber. The remaining cells on the membrane facing the basal chamber were fixed and stained with Giemsa. All cells were counted with a microscope.

### Detection of ANXA4, LAMP-2, and RHAMM proteins by western blotting

ANXA4 proteins were detected using whole-cell lysates of cancer cells or clinical samples by western blotting under reducing conditions with a murine monoclonal antibody, clone D-2 (Santa Cruz Biotechnology, Santa Cruz, CA, USA). After sodium dodecyl sulfate (SDS)-PAGE, separated proteins were transferred onto PVDF membranes and incubated with the primary antibody. Detection and visualization of the protein was performed with a conventional method by using horseradish peroxidase-labeled secondary antibodies (GE Healthcare, Buckinghamshire, UK) and chemiluminescent detection (Thermo Super Signal West Pico Chemiluminescent Substrate, Thermo Fisher Scientific, Waltham, MA, USA). As a loading control, western blot analysis for the anti-β-actin antibody (Sigma-Aldrich, St. Louis, MO, USA) was performed simultaneously. Western blotting with a mouse monoclonal anti-LAMP2 antibody (clone H4B4; EXBIO, Praha, Czech Republic) and with a rabbit monoclonal anti-RHAMM antibody (clone EPR4055; GeneTex, Irvine, CA, USA) was performed with the same system according to the manufacturer's instructions.

To detect the two ANXA4 proteins with different IEPs, 2D-PAGE followed by western blotting was performed with the Zoom IPG Runner System (Invitrogen). For cultivated cells, semi-confluent cells on a 90-mm dish were lysed in 140 µl of sample lysing buffer (7 M urea, 2 M thiourea, 4% CHAPS) with vigorous agitation. After addition of dithiothreitol (20 mM), Zoom Carrier Ampholytes 4–7 (0.5%), and bromophenol blue (0.002%), the soluble fraction was collected by centrifugation and supplemented with 0.5% N,N-dimethylacrylamide. After sample preparation, the isoelectric-focusing electrophoresis followed by PAGE separation was carried out precisely according to the manufacturer's instructions. ANXA4 on the membrane was detected as described above. Frozen cancer tissues were homogenized in RIPA buffer (25 mM Tris-HCl, pH 7.6, 150 mM NaCl, 1% Triton X-100, 1% sodium deoxycholate, 0.1% SDS) with Proteinase Inhibitor Cocktail (S3804; Sigma). After the total protein concentration was determined, samples were subjected to the 2D-PAGE analysis.

### Analysis of post-translational modifications possibly causing difference IEPs of the ANXA4 polypeptide

To estimate the involvement of phosphorylation and Ca^++^ ion conjugation in producing the two forms, cultivated OVISE cell lysates were treated with phosphatase (1000 U of Lambda Protein Phosphatase, New England Bio Labs, Ipswich, MA, USA) or 5 mM EDTA, salted out to dissolve with sample-lysing buffer, and subjected to 2D-PAGE analysis. To test for acetylation on lysine residues, OVISE cells were cultivated with 20 µM of lysine deacetylase inhibitor suberoylanilide hydroxamic acid (SAHA/vorinostat; Cayman Chemical, Ann Arbor, MI, USA), or in 3 µM of MS-275 (entinostat, Cayman Chemical) for 24 h before harvesting the cells, and subjected to 2D-PAGE followed by western blotting for ANXA4 analysis.

### IHC

The medical records from 1998 to 2009 at the Department of Gynecology, Yokohama City University Hospital were checked, and 85 surgically removed FFPE samples (52 CCC, 14 SC, 6 endometrioid adenocarcinomas, 4 mucinous adenocarcinomas, 3 poorly differentiated or undifferentiated carcinomas, 3 low malignant potential (LMP) tumors, 2 mucinous adenomas, and 3 premenopausal and 3 postmenopausal normal ovarian tissues) were subjected to ANXA4 immunohistochemistry using antibody for ANXA4 at 4 µg/ml (clone N-19, Santa Cruz Biotechnology). Immunoreactivity was visualized by the peroxidase-labeled amino acid polymer method with Histofine simple stain MAX-PO (Nichirei Co., Tokyo, Japan) according to the manufacturer's protocols. Sections were counterstained with hematoxylin. Results were evaluated using the German immunoreactive score [24] with some modification. Briefly, immunostaining intensity was rated using four ranks, from 0 (none) to 3 (intense). Numbers of immunoreactive cancer cells were also estimated in four grades as 0 (none), 1 (1–10% cells per field), 2 (10–50%), or 3(> 50%) [25]. Two independent examiners scored each case and the average of their scores was used as the IHC score for the case in this analysis.

### Statistical analysis

For analysis of results, the *t*-test was performed. Results are shown as means (± standard deviation). *P*<0.05 was considered statistically significant.

## Results

### ANXA4 was highly expressed in CCC specimens

**Figure 1:**
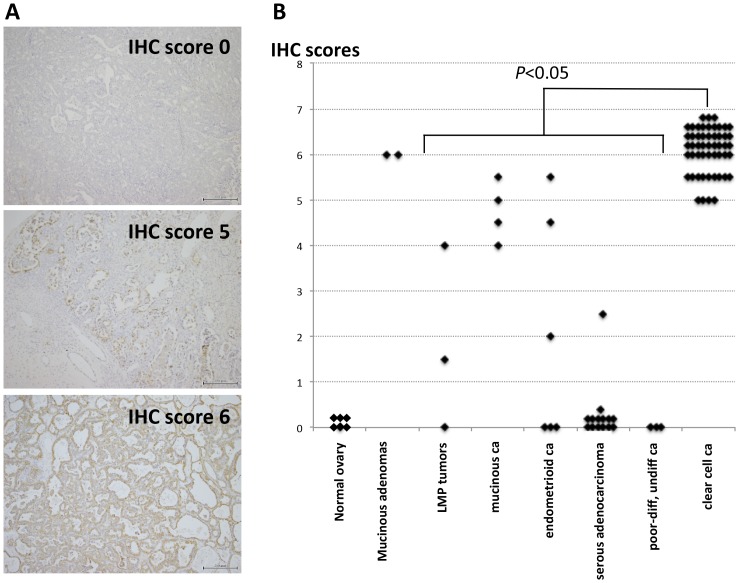
ANXA4 expression visualized in surgically removed ovarian tumors using IHC. (A) Representative images for ANXA4 immunohistochemical (IHC) scores are shown. Each scale bar represents 200 µm. (B) IHC scores were significantly high in clear cell carcinoma compared with tumors with low malignant potential (LMP) and carcinomas (*P*<0.05). ca, carcinoma; diff, differentiated.

Images corresponding to each representative IHC score and a summary of the results are shown in Figure 1A and B. All 52 CCC specimens demonstrated significant staining for ANXA4 (IHC score 5 or 6) without exception, with *P*<0.05 against all other types of ovarian carcinomas and LMP tumors (Figure 1B). In carcinoma cases, although few cases were available for IHC, all four mucinous carcinomas and two of six endometrioid carcinomas showed high levels (IHC score 4 or greater) of ANXA4 expression. Conversely, all three poorly or undifferentiated carcinomas and 13 of 14 SC expressed no detectable ANXA4. Only two mucinous adenomas were examined, but both of them showed an IHC score 6 expression of ANXA4, presenting a similarity to their malignant counterpart. Only one case of normal premenopausal ovary was available for IHC, but no ovarian components, including surface epithelium, follicles, and stromal tissues, were reactive for ANXA4.

### Establishment and growth properties of ANXA4 KO CCC cell lines

**Figure 2:**
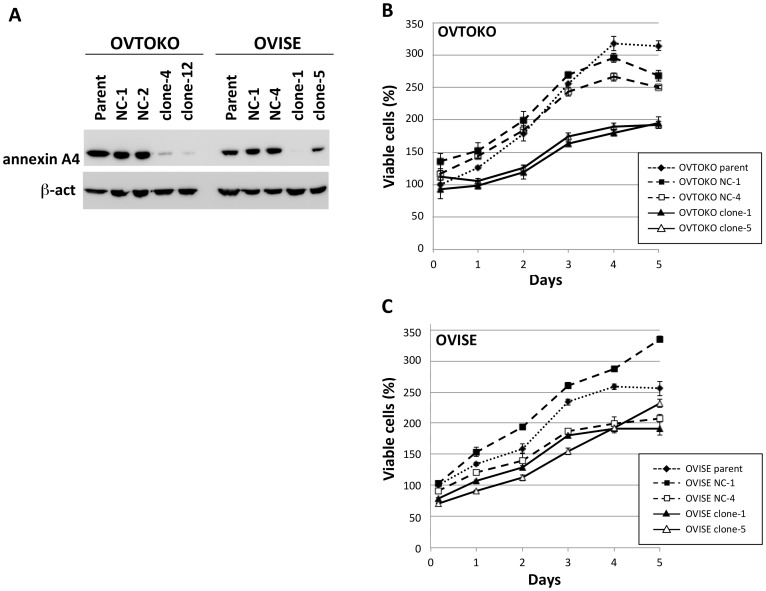
Establishment of ANXA4 knockdown clones and the effect on cell proliferation. (A) Western blotting for ANXA4, with β-actin as a loading control. Negative control clones, expressing non-*ANXA4* mRNA-targeting shRNA are designated as NC [clone number] with data for parental cells. (B, C) Proliferation was examined by a WST-1 assay as described in the text. Experiments for clones and parental cells were performed in triplicate.

We reported previously that two ANXA4 polypeptides are identified in 2D-PAGE with almost the same molecular weight but with different IEPs [10]. OVTOKO cells predominantly express an ANXA4 with a more acidic IEP (hereafter, acidic ANXA4), but show scant expression of the ANXA4 with a more basic IEP (hereafter, basic ANXA4). Conversely, OVISE cells predominantly express basic ANXA4 but show detectable amounts of acidic ANXA4. Therefore, we chose these two cell lines to establish ANXA4-KO (KO) counterparts. With introduction of plasmids that expressed shRNA to target *ANXA4* mRNA or non-targeting controls, followed by selection with G-418, we obtained two control cell lines and two cell lines with barely detectable or significantly attenuated ANXA4 expression for both OVTOKO and OVISE (Figure 2A). Using established cells together with the parent cells, we first evaluated the effect of ANXA4 KO on cellular growth. ANXA4 KO-OVTOKO cell lines showed significant growth suppression with doubling times ∼250% longer than for their control or parental cells (Figure 2B). ANXA4 KO-OVISE cell lines also showed growth suppression, with doubling times ∼200% longer than for control or parental cells. However, another control cell line, OVISE NC-4, showed suppressed growth similar to KO cells (Figure 2C).

### Effect of ANXA4 KO on chemo-sensitivity to carboplatin and paclitaxel

**Figure 3:**
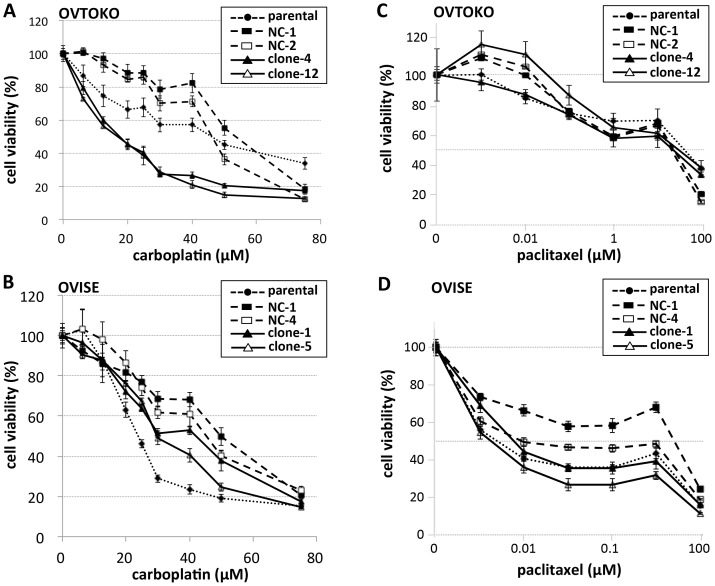
Effect of ANXA4 knockdown on carboplatin and paclitaxel sensitivity. Sensitivity of OVTOKO cell to carboplatin (A) and paclitaxel (C); and of OVISE cells to carboplatin (B) and paclitaxel (D). Drug concentrations are indicated below each panel; cell viability under each concentration was evaluated by a WST-1 assay as described in the text. Experiments for clones and parental cells were performed in triplicate.

At first, parental OVTOKO and OVISE cells tended to show higher sensitivities to both carboplatin and paclitaxel when compared with the corresponding negative control cell lines that expressed non-targeting shRNA (Figure 3). This might be caused by G-418 selection through establishment of these cell lines; therefore, we compared ANXA4-KO cell lines with their negative control cell lines. ANXA4 KO-OVTOKO cells showed significantly enhanced sensitivity to carboplatin; their IC_50_ was less than 40% that of control cells (Figure 3A). ANXA4 KO-OVISE cells also showed increased sensitivity to carboplatin when compared with control cells, but not as significantly as with OVTOKO cells (Figure 3B).

OVTOKO cell lines showed inherent resistance to paclitaxel, with IC_50_ greater than 50 µM, compared with OVISE cells, whose IC_50_s were less than 10 µM except for a negative control line, NC-1 (Figure 3C and D). We found no significant changes between ANXA4 KO-OVTOKO cells and their controls in sensitivity to paclitaxel (Figure 3C). For OVISE cells, the two non-targeting control cell lines showed different sensitivities to paclitaxel; ANXA4 KO-OVISE cells showed greater sensitivity to paclitaxel than did control cells (Figure 3D).

### Effect of ANXA4 KO on migration and invasion

**Figure 4:**
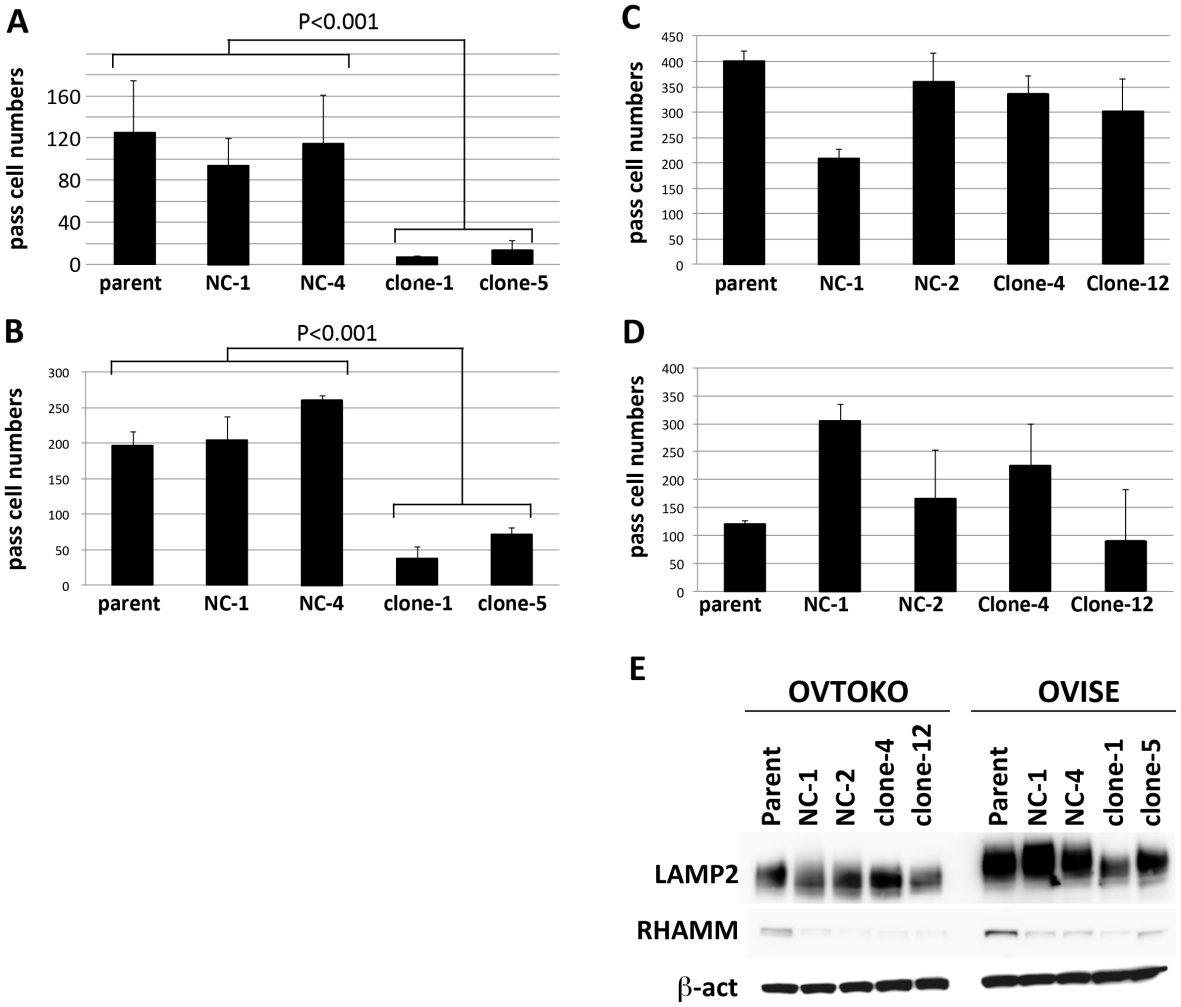
Effect of ANXA4 knockdown on cell migration and invasion and western blotting for membrane proteins RHAMM and LAMP2. OVISE cell migration (A) and invasion (B); and OVTOKO cell migration (C) and invasion (D). Experiments for clones and parental cells were performed in triplicate. (E) Expression of membrane proteins RHAMM and LAMP2 was demonstrated by western blotting in OVISE and OVTOKO cells. β-actin was used as a loading control in western blotting.

ANXA4 KO-OVISE cells demonstrated significantly decreased migration and invasion capability in the transwell assay when compared with parental and control cell lines (Figure 4A and B). Conversely, ANXA4 KO-OVTOKO cells showed no significant changes in migration and invasion activity compared with parental and control cell lines (Figure 4C and D).

Expression of two membrane proteins, RHAMM and LAMP2, was examined by western blotting in OVISE and OVTOK cell lines. Expression of RHAMM was very low in parental cells and ANXA4 KO cells. Conversely, a large amount of LAMP2 was identified in OVISE parental cells and control cells, whereas this expression was attenuated in ANXA4 KO- OVISE cells. Expression of LAMP2 was not significant in OVTOKO cells nor changed by ANXA4 KO (Figure 4E).

### Demonstration of the two ANXA4 subtypes in CCC specimens and implication in patients; clinical characteristics

**Figure 5:**
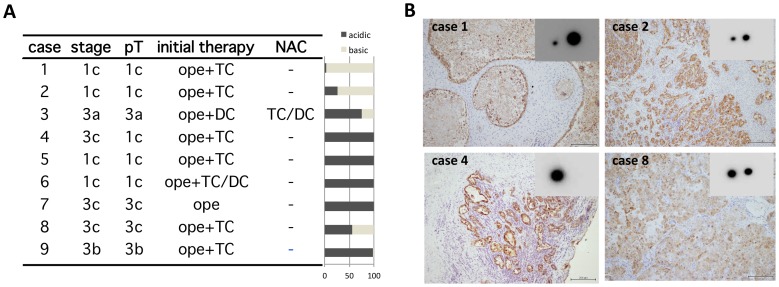
Demonstration of the two ANXA4 subtypes with different IEPs in surgically removed CCC samples. (A) Table of clinical CCC samples examined by 2D-PAGE and bar graph (on right) of their ratios of ANXA4 subtypes. pT, pathological T stage; Ope, surgical removal of the tumors; +, post-operational chemotherapy; TC, combined paclitaxel and carboplatin chemotherapy; DC, combined docetaxel and carboplatin chemotherapy; NAC, neoadjuvant chemotherapy. (B) Representative images of 2D-PAGE followed by western blotting with a IHC image of the corresponding case (scale bar: 200 µm).

To test whether we could distinguish the two ANXA4 subtypes in clinical samples as we did in established cell lines, we used nine samples from patients with CCC from whom frozen tumor tissues of their primary surgeries were available (Figure 5A). At first, we evaluated ANXA4 IHC images and confirmed that the annex A4 proteins in the tissues were located predominantly in tumor cells, rather than non-neoplastic components, such as stromal cells, infiltrating inflammatory cells, or blood cells in vessels (representative images in Figure 5B). Using 2D-PAGE followed by western blotting, we identified both acidic and basic ANXA4 proteins in all clinical CCC samples; ratios of the subtypes varied by patient (Figure 5A and B).

Of the five patients with stage 3 CCC whose samples we examined, two still had measurable tumors after their first surgery and received combined carboplatin and paclitaxel chemotherapy (TC) after their surgeries (cases 8 and 9 in Figure 5B). Therefore, we could directly evaluate the effect of TC on these tumors. Response to chemotherapy was poor in these patients, with progressive disease for case 8 and partial response for case 9. Except for the two stage 1c cases (cases 1 and 2), most samples contained the acidic ANXA4 predominantly; in cases 4, 5, 6, and 7, especially, the basic form was barely detectable.

### Investigation of a post-translational modification that might cause the two ANXA4 subtypes

**Figure 6:**
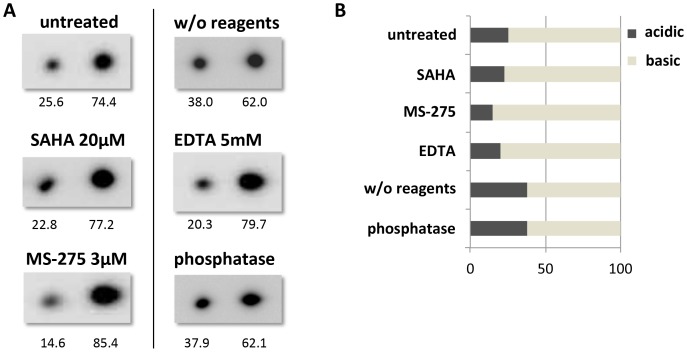
Involvement of phosphorylation, Ca^2+^ conjugation, and acetylation in production of two ANXA4 subtypes with different IEPs. (A) OVISE cells containing both acidic and basic ANXA4 subtypes were used to study effects of deacetylation, Ca^2+^ chelation, and dephosphorylation on subtype ratios. Each 2D-PAGE and western blot image for ANXA4 shows the treatment (above the panel), and the signal intensity (below each spot), as evaluated by an image analyzer ImageQuant LAS 4000 (GE Healthcare Life Science, UK). The left side spot indicates the acidic form and the right side spot indicates the basic form of ANXA4 respectively. (B) Acidic/basic subtype ratios were derived from signal intensities, and are shown in a bar of 100%.

To elucidate what causes the IEP difference without any evident change in the molecular weight on the 2D-PAGE, we interrogated the involvement of phosphorylation, Ca^2+^ conjugation, or acetylation on the lysine residues. Chelating bivalent cations by EDTA treatment or removal of phosphorylation by phosphatase treatment did not change the proportion of acidic and basic annexin spots on 2D-PAGE of OVISE cell lysates, nor did cultivation in the presence of two different deacetylase inhibitors, SAHA and MS-275 (Figure 6A and B).

## Discussion

Although *ANXA4* is well known as an CCC signature gene and expression profiles of CCC cell lines and clinical samples have been reported in several studies, as far as we know, no reports on ANXA4 function directly in CCC cells have been published. In the present study, we first showed ANXA4 to affect proliferation, chemoresistance, and migration and invasion of CCC cells *in vitro*. We used two CCC cell lines that both express significant amounts of ANXA4 protein, one of which, OVTOKO, contains exclusively acidic ANXA4, whereas the other, OVISE, contains both forms but predominantly basic ANXA4. Biological phenotypes examined were differentially affected by ANXA4 KO in these cells.

We first reconfirmed the abundant and significant expression of ANXA4 in CCC against other types of ovarian carcinomas in clinical samples, using IHC as in two preceding reports [11], [12]. Mucinous adenocarcinomas also showed high expression of ANXA4, as did their benign counterpart, mucinous adenomas. Although some SC cases reportedly express considerable amounts of ANXA4, linking them to chemoresistance [11], [20], we did not find such significant expression in the SC cases we examined here.

ANXA4 KO significantly decreased proliferation of OVTOKO CCC cells. Although one of the negative control cell lines showed growth suppression similar to the KO cells, we considered that OVISE cell proliferation tended to be down-regulated by ANXA4 KO. Only one study was found in the literature on the relationship between cell proliferation and ANXA4 in cancer cells; Lin and colleagues recently found that ANXA4 overexpression or attenuation by siRNA in a gastric adenocarcinoma cell line, AGS, indicated its function to be a growth activator [16]. Further analysis of changes in gene expression caused by ANXA4 overexpression showed that activation of cyclin-dependent kinase 1 (CDK1) and AKT, together with suppression of p21, were apparently involved in the growth activation [16]. The study began from the identification of ANXA4 as an up-regulated protein in *Helicobacter pylori*-infected gastric cancer [26]. Because CCC is also associated with persistent inflammation caused by endometriosis [27], similarity between CCC and *H. pylori*-associated gastric cancer is of interest. Conversely, there are several studies demonstrating involvement of other types of annexins, such as annexins A1 and 2, in proliferation of other types of cancer cells [28], [29]; therefore, this growth-activating property of ANXA4 might be a common feature among annexins. Unlike the high mutation rate in the HGSC, the mutation rate of the *TP53* gene in CCC is rare [1]. We have recently reported that OVTOKO and OVISE cells do not have the *TP53* mutation, and wild-type p53 enhances *ANXA4* gene expression in these cells *in vitro* [30]. This relationship between p53 and ANXA4 apparently contradicts the ANXA4 effect on proliferation and chemoresistance described later. Further precise investigation is needed on this issue.

Kim and colleagues demonstrated clearly by expressing ANXA4 in an *ANXA4*-null SC cell that expression of ANXA4 confers resistance to carboplatin and decreases intracellular platinum accumulation [11]. They speculated that ANXA4 affects enhancement of cellular platinum efflux. In our study, ANXA4 KO cells showed significantly increased sensitivity to carboplatin especially in OVTOKO cells, which may directly support the clinically observed resistance to platinum-based chemotherapy in CCC. For paclitaxel, another anticancer agent used in ovarian cancer chemotherapy, Han et al. reported that ANXA4 expression was induced in a lung cancer cell line by paclitaxel treatment *in vitro*, and overexpression of ANXA4 in 293T cells conferred paclitaxel resistance [22]. We did not observe any significant effects of ANXA4 KO on paclitaxel resistance in OVTOKO cells, although OVISE cells seemed to be slightly affected. Paclitaxel sensitivity might be controlled by a different mechanism from that of carboplatin, and this control might vary by cell type. CCC's inherent chemoresistance is sometimes thought to relate to its clinically observed slow-growth property, but ANXA4 had a growth-activating effect together with chemoresistance *in vitro*. The concept may need to be reconsidered.

ANXA4 KO resulted in significant inactivation of OVISE cell migration and invasion. Zimmerman and colleagues reported that overexpression of ANXA4 in MCF-7 breast cancer cells accelerated migration *in vitro*, which is consistent with our result on OVISE cells [21]. Conversely, ANXA4 KO showed no effect on OVTOKO cell migration and invasion. Because the ANXA4 expression in AGS gastric cancer cells induces expression of membrane proteins RHAMM and LAMP2 [16], we examined the expression of these proteins in OVISE and OVTOKO cells. RHAMM is a hyaluronan-mediated motility receptor, known to be involved in migration [31], [32]. LAMP2 has been reported as an adhesive glycoprotein that participates in the processes of invasion and metastasis of melanoma, colon cancer, or fibrosarcoma cells [33]. Although RHAMM was barely detectable in both OVISE and OVTOKO cells, a large amount of LAMP2 was identified in OVISE cells, and its expression was significantly decreased by ANXA4 KO. Because the basic ANXA4 is predominantly expressed in OVISE cells and barely detectable in OVTOKO cells, one could speculate that the basic ANXA4 may be responsible for LAMP2 expression in CCC cells. This may partly explain the reason why the significant effect of ANXA4 KO on migration and invasion was observed only in OVISE cells. Further studies of, for example, LAMP2 KO in OVISE cells, are needed to clarify this interaction.

Some phenotypic changes observed in ANXA4 KO differed between OVTOKO and OVISE cells, especially in carboplatin resistance and migration and invasion. One could speculate that the difference in subtypes of ANXA4 expressed in these cells affects these phenotypic changes, for example, in a manner that only the basic ANXA4 was involved in cell migration and invasion. We confirmed in this study that both acidic and basic subtypes of ANXA4 found in cultivated cells are actually found in clinical CCC samples in varying ratios to each other. Because only two available cases offered any light on sensitivity to chemotherapy as residual tumors after the initial operation with known content of ANXA4 subtypes, clinical significance of the subtypes is still unclear. Although we failed to find the mechanism that produces the IEP difference of ANXA4 subtypes *in vitro* by focusing on phosphorylation, lysine acetylation, or Ca2^+^ ion conjugation in the present study, further studies with proteomic approaches to reveal post-translational modification of ANXA4 should be important.

In summary, we demonstrated the involvement of ANXA4 in various kinds of biological phenotypes of CCC *in vitro*, such as proliferation, chemoresistance, and migration and invasion of CCC cells. Our study raised a possibility that the two different subtypes of ANXA4 with different IEPs could have different functions in some cell biology. Investigation of the mechanism to produce the subtypes may facilitate understanding of the function of ANXA4 in CCC and improve treatment strategies and prognoses of CCC patients.
